# The mesenchymal compartment in myelodysplastic syndrome: Its role in the pathogenesis of the disorder and its therapeutic targeting

**DOI:** 10.3389/fonc.2023.1102495

**Published:** 2023-01-25

**Authors:** Charalampos G. Pontikoglou, Angelos Matheakakis, Helen A. Papadaki

**Affiliations:** ^1^ Department of Hematology, School of Medicine, University of Crete, Heraklion, Greece; ^2^ Haemopoiesis Research Laboratory, School of Medicine, University of Crete, Heraklion, Greece

**Keywords:** hematopoiesis, bone marrow, mesenchymal stromal cells, myelodysplastic syndromes, iron overload, hypomethylating agents, lenalidomide, luspatercept

## Abstract

Myelodysplastic syndromes include a broad spectrum of malignant myeloid disorders that are characterized by dysplastic ineffective hematopoiesis, reduced peripheral blood cells counts and a high risk of progression to acute myeloid leukemia. The disease arises primarily because of accumulating chromosomal, genetic and epigenetic changes as well as immune-mediated alterations of the hematopoietic stem cells (HSCs). However, mounting evidence suggests that aberrations within the bone marrow microenvironment critically contribute to myelodysplastic syndrome (MDS) initiation and evolution by providing permissive cues that enable the abnormal HSCs to grow and eventually establish and propagate the disease. Mesenchymal stromal cells (MSCs) are crucial elements of the bone marrow microenvironment that play a key role in the regulation of HSCs by providing appropriate signals *via* soluble factors and cell contact interactions. Given their hematopoiesis supporting capacity, it has been reasonable to investigate MSCs’ potential involvement in MDS. This review discusses this issue by summarizing existing findings obtained by *in vitro* studies and murine disease models of MDS. Furthermore, the theoretical background of targeting the BM-MSCs in MDS is outlined and available therapeutic modalities are described.

## Introduction

**Figure 1:**
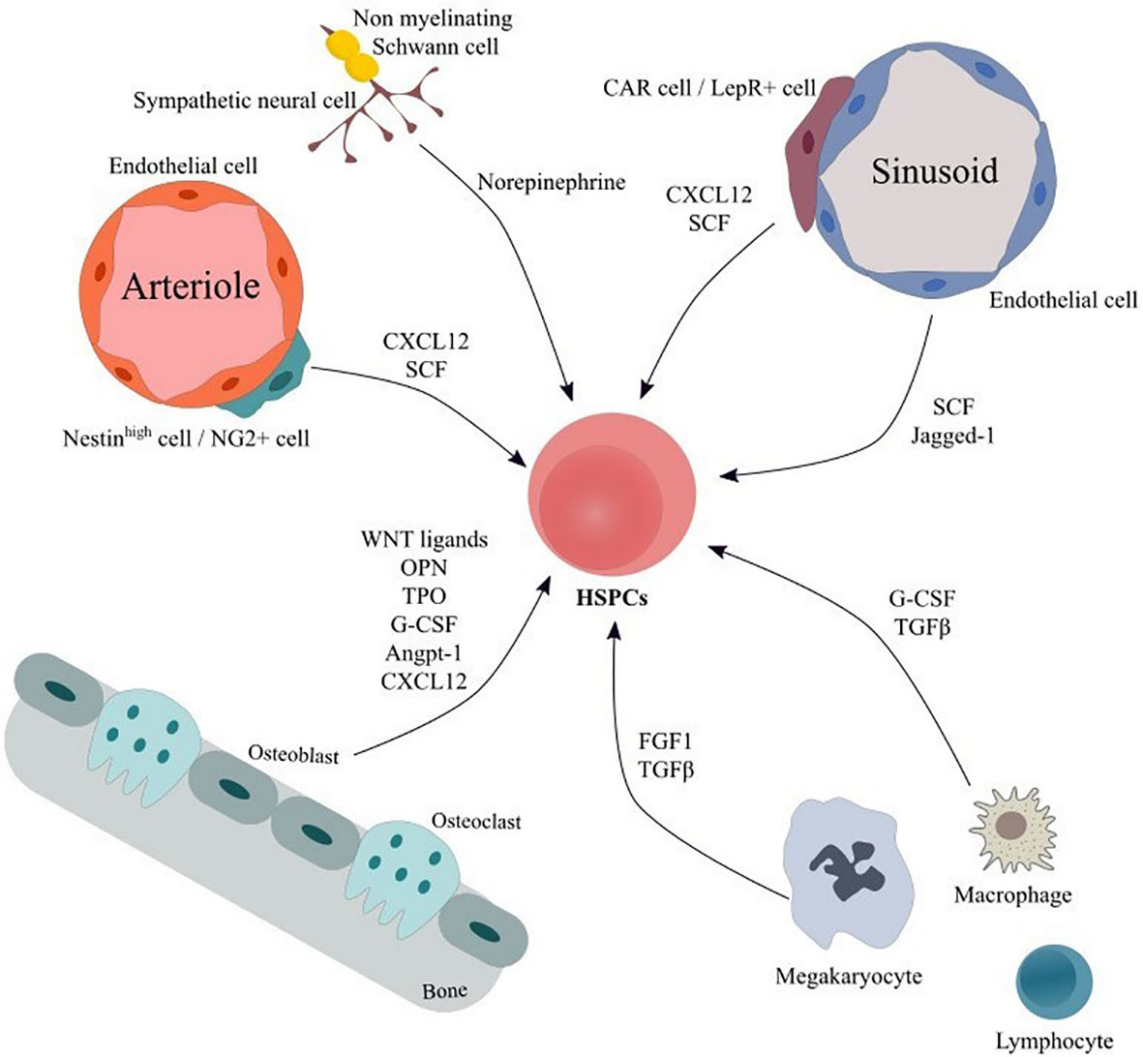
Main cellular components and soluble factors of hematopoietic HSC niche. The bone marrow niche comprises a variety of cellular populations embedded in the extracellular matrix. Cells of hematopoietic origin including macrophages, megakaryocytes and lymphocyte subgroups interact both *via* cell-to-cell connection and *via* secretion of soluble factor thus modulating HSPCs’ proliferation, differentiation and activation. ECs form a vast net of arterioles, sinusoids and capillaries and contribute in HSPC support by secreting factors, such as SCF and Notch ligands. MSC subpopulations modulate HSPCs’ maintenance, retention and proliferation and can differentiate to osteoblasts, adipocytes and chondrocytes. In addition, sympathetic neuronal cells act on HSPCs *via* adrenergic signaling, thereby inducing hematopoietic stem cells’ egress from the BM. Finally, Schwann cells may regulate hematopoietic stem cells’ quiescence. Angpt-1, Angiopoietin-1; CAR cells, CXCL12-abundant reticular cells; CXCL12, C-X-C motif chemokine ligand 12; ECs, endothelial cells; FGF1, fibroblast growth factor 1; G-CSF, granulocyte-colony stimulating factor; HSPCs, Hematopoietic stem and progenitor cells; LepR+ cell, leptin receptor+ cell; MSC, mesenchymal stromal cells; NG2+ cells, neural–glial antigen 2+ cells; OPN, Osteopontin; SCF, stem cell factor; TGFβ, transforming growth factor beta; TPO, Thrombopoietin.

Within the adult Bone Marrow (BM) hematopoiesis take place in specialized microenvironments called niches that regulate the balance between quiescence, proliferation, differentiation and self-renewal of hematopoietic stem and progenitor cells (HSPCs) (1). The BM niche is composed of a network of cells of hematopoietic and non-hematopoietic origin as well as of extracellular matrix, which provide the structural support, the physical interactions and the molecular cues for hematopoietic stem cell (HSC) maintenance and function (1). Cells of hematopoietic lineage include lymphocytes, macrophages, osteoclasts, megakaryocytes as well as myeloid derived suppressor cells (MDSCs), while non-hematopoietic components comprise mesenchymal stromal cells (MSCs) and their progeny, vascular endothelial cells (ECs), fibroblasts, sympathetic neurons and non-myelinating Schwann cells collectively contributing to BM homeostasis (**Figure 1**). For a more detailed discussion of the niche the reader is referred to some excellent reviews (1–4).

As far as BM-MSCs are concerned, they consist of a minor and a heterogeneous population of perivascular cells with broad immunoregulatory properties that have the potential to differentiate into osteoblasts, chondrocytes and adipocytes and support hematopoiesis (1–3) (5). Using appropriate mouse models, various BM-MSC subsets with partially overlapping characteristics have been identified, such as CXCL12-abundant reticular (CAR) cells, leptin receptor (LepR)^+^ cells, nestin^+^ cells and neural–glial antigen 2 (NG2)^+^ cells (6–8) (**Figure 1**). Nestin^bright^ and NG2^+^ cells are associated with arterioles, whereas CAR cells, LepR^+^ cells and nestin^dim^ cells are localized around sinusoids (9, 10). The aforementioned BM-MSC subpopulations are involved in the maintenance, proliferation, and retention of HSCs (1–3).

Due to the low frequency of MSCs within the BM, data regarding their impact in regulating the homeostasis of HSCs is mainly derived from studies using ex vivo-expanded cells. The latter are defined based on the three minimal criteria established by the International Society for Cellular Therapy (ISCT) (11) i.e. (a) adherence to plastic, (b) expression of the surface antigens CD73, CD90, CD105 while lacking the expression of the hematopoietic and endothelial molecules CD11b, CD14, CD19, CD34, CD45, CD79a, CD11b and HLA-DR, and (c) *in vitro* differentiation into three mesodermal lineages (osteoblasts, adipocytes, chondrocytes).

As the crucial role of BM-MSCs and their progeny in the control of hematopoiesis is increasingly being acknowledged (12, 13), it may be reasonable to consider the possibility that defects of these cell populations are involved in the establishment and/or propagation of hematological malignancies. Myelodysplastic syndromes represent an attractive disease model to investigate this hypothesis.

Myelodysplastic syndromes consist of an heterogeneous group of clonal hematological disorders characterized by ineffective dysplastic hematopoiesis, peripheral blood cytopenias and an increased risk of transformation into acute myeloid leukemia (AML) (14). Patients with Myelodysplastic Syndrome (MDS) may be largely asymptomatic with mild cytopenias and long-life expectancy or they may exhibit profound symptoms, significantly reduced blood counts, and a very poor prognosis. The former are classified under the lower-risk (LR) group, whereas the latter under the higher-risk (HR) group, based on the International Prognostic Scoring System (IPSS) categorization (14). Notably, these groups differ in disease pathogenesis, risk of disease progression and survival and are managed with different therapeutic modalities (14). The pathogenesis of MDS development and disease progression to AML has long been recognized to involve accumulation of cytogenetic, genetic and epigenetic aberrations as well as immune-mediated alterations of hematopoietic cells (14). However, histologic studies in the 90s had already provided evidence for abnormalities in the components of the BM microenvironment and alterations in the localization of hematopoietic cells within the BM [reviewed in (15)]. These preliminary findings suggested that MDS should not merely be considered as a disorder of the hematopoietic compartment, but rather of the whole tissue.

**Figure 2:**
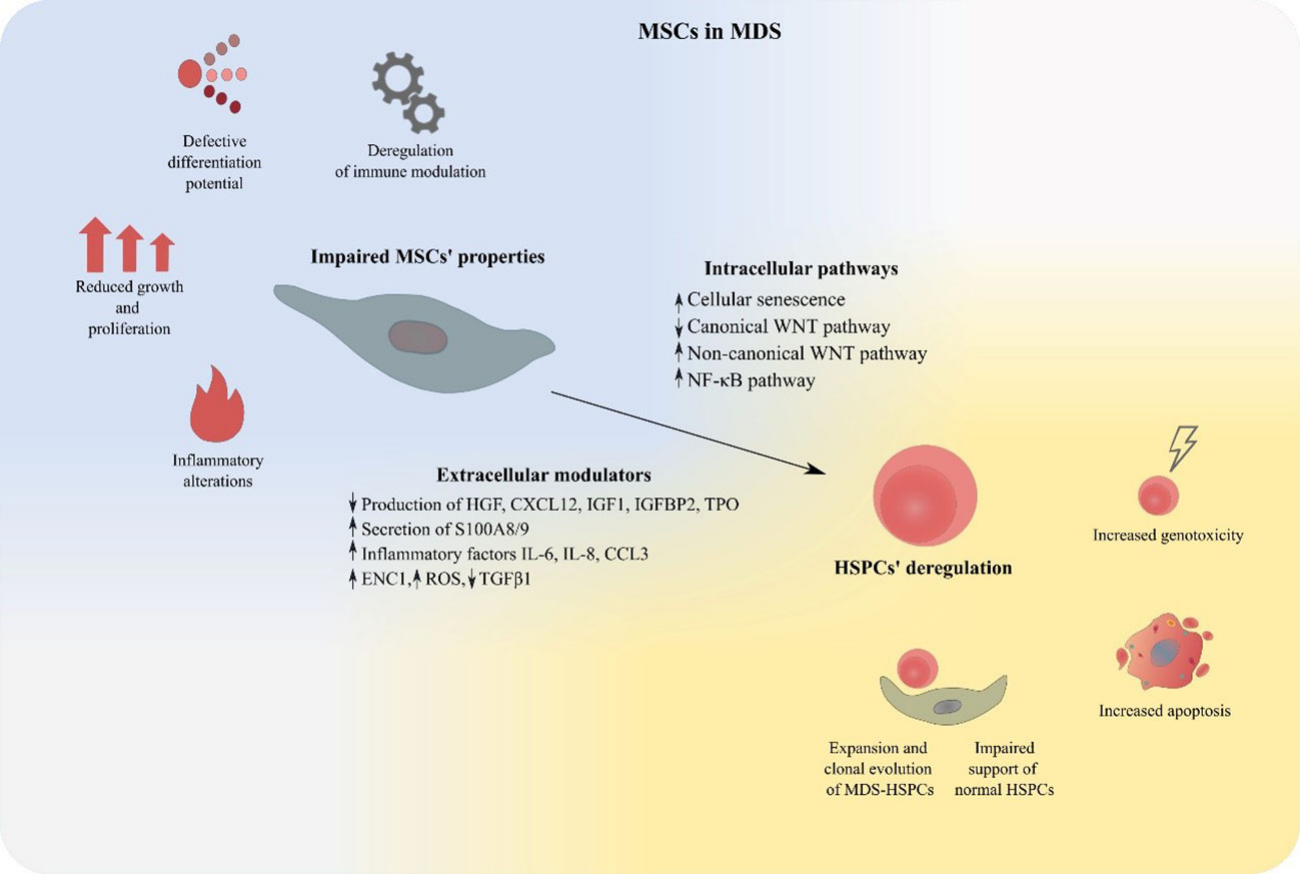
MSCs deregulation in MDS. MDS-derived BM-MSCs exhibit impaired properties regarding their proliferation, differentiation, modulation of the immune system and support of hematopoiesis. Expansion of malignant hematopoietic cells, suppression of normal HSPCs, increased apoptosis and genotoxicity are features that characterize MSC-mediated HSPC deregulation in MDS. BM-MSCs, Bone Marrow Mesenchymal Stromal Cells; HSPCs, Hematopoietic stem and progenitor cells; MDS, MDS Myelodysplastic Syndrome.

In the present review, we will attempt to summarize existing knowledge provided by *in vitro* studies and mouse models supporting the notion of an impaired BM-MSC compartment in MDS and discuss its contribution in the pathogenesis of the disorder. Furthermore, the theoretical background of BM-MSCs’ therapeutic targeting in MDS will be outlined and an overview of relevant *in vitro* data will be provided (**Figure 2**).

## Properties of *ex vivo* expanded MDS-derived BM-MSCs

### Impaired morphology, proliferation and differentiation potential of patient-derived BM-MSCs

Early studies investigating the properties of MDS-derived BM-MSCs were conducted in *ex vivo* expanded cells, as their *in situ* counterparts represent only a minor fraction of BM nucleated cells (16). Published data are at times conflicting and this might be due to the variability of experimental approaches and to patient heterogeneity (16). Within this context, cultured MDS-derived BM-MSCs have been reported to exhibit an irregular morphology (**Table 1**) (17–22, 40), whereas other studies (**Table 1**) found no morphological abnormalities in patient BM-MSCs as compared to their normal counterparts (23–30). Moreover, most studies (**Table 1**) suggest that patient BM-MSCs do not differ in terms of immunophenotype from MSCs derived from healthy donors (25–30), although reduced expression of CD90, CD104 and CD105 has also been observed (17, 41).

**Table 1:** *In vitro* studies of BM-MSCs in MDS.

Reference	Study Population, Median Age years (range)	Major Findings
(17)	36 patients, 70 (34–89) 15 HDs, 57 (37–88)	*Ex vivo* expanded patient-derived BM-MSCs exhibited a more thick and granular morphology and a lower expression of CD105 and CD104 and had defective growth potential as compared to HD-derived BM-MSCs. Genomic aberrations (mainly gains) in 17/17 tested MDS-derived BM-MSCs; not present in hematopoietic cells. No difference in cell cycle distribution, apoptosis, osteoblastic and adipocytic differentiation capacity between patient- and HD-derived BM-MSCs. Impaired chondrocyte differentiation of MDS-derived BM-MSCs
(18)	20 patients, 68 (32–68) 6 HDs, 60 (56–65)	*Ex vivo* expanded patient-derived BM-MSCs had altered morphology and impaired osteogenic and adipogenic differentiation potential. They were more senescent and had defective proliferation and clonality, as compared to HD-derived BM-MSCs. MDS-derived BM-MSCs demonstrated diverse expression of cell adhesion molecules and reduced expression of ANG1, SCF, CXCL12. Moreover, they exhibited defective hematopoietic support of CAFCs and clonal hematopoietic progenitors. Incubation of MDS-derived BM-MSCs with lenalidomide decreased CAFCs whereas it increased the formation of erythroid and myeloid colonies
(19)	121 patients, 66 (21–91) 67 HDs, 63 (31–85)	*Ex vivo* expanded patient-derived BM-MSCs were larger and disorganized, more senescent and had defective growth potential as compared to HD-derived BM-MSCs. MDS-derived BM-MSCs had reduced osteogenic potential, impaired expression of chemokines and molecules involved in hematopoiesis and defective hematopoietic support, such as Osteopontin, Jagged1, Kit-ligand and Angiopoietin as well as several chemokines. Patient and HD-derived BM-MSCs had differential methylation patterns showing enrichment for biological processes associated with cellular phenotypes and transcriptional regulation.
(20)	30 patients, 72 (48–85) 32 HDs, 67 (27–77)	MDS-derived BM-MSCs exhibited reduced proliferative capacity as compared to their normal counterparts, not attributed to increased replicative senescence. They also displayed increased expression of genes related to the noncanonical WNT pathway along with downregulation of genes related to the canonical WNT pathway and upregulation of canonical WNT inhibitors. Patient-derived BM-MSCs had normal differentiation potential but defective osteogenic and adipogenic lineage priming under non-differentiating culture conditions. Pharmacological activation of canonical WNT signaling in patient BM-MSCs led to an increase in cell proliferation and upregulation in the expression of early osteogenesis-related genes. MDS-derived BM-MSCs exhibited impaired capacity to support normal CD34^+^ myeloid and erythroid colony formation which was hypothesized to be associated with the increased Jag*ged1* expression
(21)	11 LR patients, 76 (33–84) 10 HR patients, 61.5 (33–87) 6 HDs, 46 (35–49)	*Ex vivo* expanded patient-derived BM-MSCs had irregular morphology, reduced proliferation and differentiation potential, decreased expression of hematopoietic factors and increased levels of IL6. MDS-derived BM-MSCs and especially HR-MDS-derived BM-MSCs were epigenetically deregulated and supported poorly HSPCs. Treatment of patient-derived BM-MSCs with AZA reversed their functional abnormalities and improved their capacity to support hematopoietic cells for *in vivo* engraftment
(22)	20 patients, 74 (40–87) 33 HDs, 55 (20–78)	Prospectively isolated CD73^+^CD105^+^CD271^+^ BM-MSCs from MDS patients displayed significantly reduced frequency within the BM, decreased clonogenic potential and abnormal morphology during culture as compared to their normal counterparts. In MDS patients the aforementioned BM-MSC population had normal osteogenic potential but demonstrated increased adipogenic capacity with decreased expression of the adipogenic cell fate inhibitor DLk1
(23)	11 patients, 52 (17–80) 5 HDs, NR (18–42)	*Ex vivo* expanded patient-derived BM-MSCs did not differ in morphology or immunophenotype, as compared to their normal counterparts. In 5/9 patients chromosomal aberrations were detected in BM-MSCs
(24)	10 patients, 69.5 (51–90) 15 HDs, NR	*Ex vivo* expanded patient-derived BM-MSCs demonstrated normal morphology, reduced proliferation potential and osteoblastic differentiation capacity, while they retained adipogenic differentiation ability as compared to their normal counterparts. Patient-derived BM-MSCs exhibited decreased hematopoiesis supporting capacity of CAFCs
(25)	15 patients, 54 (40–84) 12 HDs, NR	*Ex vivo* expanded patient-derived FLK1^+^CD31^-^CD34^-^ BM-MSCs had normal karyotype and did not differ in morphology or immunophenotype as compared to their normal counterparts. Patient-derived BM-MSCs exhibited defective potential to inhibit T lymphocyte proliferation and activation
(26)	12 patients, NR (35–58) 9 HDs, NR (17–41)	*Ex vivo* expanded patient-derived BM-MSCs did not differ in morphology or immunophenotype as compared to their normal counterparts. However, they had impaired capacity to inhibit T cell proliferation
(27)	16 patients, NR 6 HDs, NR (18–42)	*Ex vivo* expanded patient- and HD-derived BM-MSCs did not differ in morphology, immunophenotype, differentiation capacity and hematopoietic support of umbilical cord blood progenitors. Patient-derived BM-MSCs had abnormal karyotype (67%) and exhibited higher expression of IL1β, SCF following TNFα stimulation and increased expression of CD49b
(28)	14 LR patients, NR (38–56) 15 HR patients, NR (32–57) 10 HDs, NR (30–55)	*Ex vivo* expanded patient- and HD-derived BM-MSCs did not differ in morphology or immunophenotype. MDS derived BM-MSCs secreted more IL6, but less TGFβ1 and HGF, as compared to their normal counterparts. They demonstrated a weaker inhibitory effect on T cell proliferation but a similar capacity to induce Tregs, in comparison to normal BM-MSCs. LR MDS secreted less TGFβ1 (shown to be involved in Treg generation), had a lower Treg inducible rate and exerted a poorer down regulation of T cell proliferation and as compared to HR MDS
(29)	14 patients, 39 (32–52) 8 HDs, NR (21–49)	*Ex vivo* expanded patient- and HD-derived BM-MSC clones did not differ in morphology, immunophenotype, growth potential and differentiation capacity. Patient-derived BM-MSCs had normal karyotype, impaired expression of hematopoietic cytokines, support of hematopoiesis and defective inhibition of T cell activation and proliferation compared to their normal counterparts
(30)	26 MDS, 78 (53–90) 12 HDs, 42 (19–62)	*Ex vivo* expanded patient- and HD-derived BM-MSCs did not differ in morphology, immunophenotype differentiation potential support of leukemic cell viability and proliferation and Treg cell induction. Patient-derived BM-MSCs displayed decreased frequency within the BM, reduced proliferative capacity and not the cytogenetic abnormalities of hematopoietic cells
(31)	13 patients, 70 (60–84) 20 HDs, NR	*Ex vivo* expanded MDS-derived BM-MSCs displayed reduced clonogenic and proliferative potential but did not differ in terms of differentiation capacity or inhibition of T cell proliferation as compared to their normal counterparts. The production of TNFα, IL1β, IL6, VEGF, CXCL12 did not differ between patient and HDs BM-MSCs. Cultured MDS-derived BM-MSCs did not harbor the cytogenetic abnormalities present in hematopoietic cells but in 4/13 cases developed irrelevant chromosomal alterations (trisomies 5 and 7)
(32)	20 patients, 73 (NR) 8 HDs, 63 (NR)	Decreased growth potential of STRO-1^+^CD73^-^ and STRO-1^-^ CD73^+^ subpopulations isolated from cultured MDS-derived BM-MSCs as compared to those isolated from HD-derived MSCs. Growth impairment of MDS-derived BM-MSCs was associated with reduced expression of CD44 and CD49e
(33)	98 patients, NR (44–86) 28 HDs, NR (36–84)	*Ex vivo* expanded patient derived BM-MSCs exhibited genotoxic stress markers, senescence markers and increased expression of inflammatory genes. As compared to HD-derived BM-MSCs, patient-derived BM-MSCs carried a larger number of mutations with an overall higher VAF and displayed distinct mutational signatures. Detected mutations were not found in non-expanded sorted BM-MSCs from the same patients
(34)	12 patients, 62 (17–76) 2 HDs, NR	*Ex vivo* expanded patient-derived BM-MSCs did not differ in growth potential and osteoblastic differentiation capacity as compared to their normal counterparts. MDS-derived BM-MSCs did not carry the chromosomal abnormalities detected in HSCs by FISH. Patient-derived BM-MSCs were able to promote the growth of autologous clonal progenitors and to support LTC-IC derived progeny
(35)	30 patients, 72 (44–92) 27 HDs, 40 (21–65)	BM-MSCs of LR MDS patients secrete extracellular vesicles with a different cargo than their normal counterparts extracellular vesicles are incorporated into normal CD34^+^ cells and modify their gene expression, *via* microRNA transfer (such as miR-10a and miR-15a), and increase their clonogenic potential and viability.
(36)	45 patients, NR 10 HDs, NR	BM-MSCs from LR-MDS patients exhibited activated NF-κB signaling leading to transcriptional upregulation of inflammatory molecules, including factors with a negative impact on hematopoiesis. In co-culture experiments, ex vivo expanded murine OP9 mesenchymal cells with constitutive NF-κB activation reduced normal HSPC numbers and function
(37)	16 LR patients, NR (41–65) 15 HR patients, NR (39–62) 8 HDs, NR (37–61)	MDS derived BM-MSCs did not differ in the ability to inhibit DC maturation and differentiation as compared to their normal counterparts. However, MDS-derived BM-MSCs had decreased capacity than to inhibit DC endocytosis, to induce IL12 secretion and to suppress DC mediated T cell proliferation. These effects were partly attributed to TGFβ1 derived from patient MSCs. The effects of LR MSCs on the differentiation, maturation and function of DCs were weaker as compared to higher risk MDS
(38)	10 patients, 66 (13–72) 11 HDs, 60 (43–84)	MDS-derived BM-MSCs exhibited increased expression of the ROS pathway regulator gene *ENC1* thereby resulting in down-regulation of the TGF-β repressor gene MAB21L2 in monocytes. These MDS-MSC–conditioned monocytes exerted NK and T cell function inhibition.
(39)	13 patients, 70 (34–91) number of HDs NR, 45 (35–61)	*Ex vivo* expanded MDS-derived BM-MSCs demonstrated aberrant DNA hypermethylation. This was abrogated following treatment with 5-AZA and was further associated with improved support of erythropoiesis. The WNT pathway antagonist FRZB was shown to be hypermethylated and down-regulated in both cultured as well as primary non-expanded MDS-derived BM-MSCs. This down-regulation could lead to b-catenin activation in HSCs co-cultured with patient stroma. WNT activation signature was also detected in advanced MDS cases and was associated with adverse prognosis. In line with these findings, in a murine model of MDS constitutive WNT activation resulted in lethal myeloid disease.

ANG, Angiopoietin; 5-AZA, 5-Azacitidine; BM-MSCs, Bone Marrow Mesenchymal Stromal Cells; CAFCs, cobblestone-area forming cells; CXCL12, C-X-C motif chemokine ligand 12; DC, Dendritic Cell; HD, Healthy Donor; HGF, Hepatocyte growth factor; HR, Higher risk; HSPCs, Hematopoietic stem/progenitor cells; IL, Interleukin; LR, Lower risk; LTC-IC, long-term culture initiating cell; MAB21L2, Mab-21 Like 2; MDS, Myelodysplastic Syndrome; NF-κB, Nuclear factor kappa light chain enhancer of activated B cells; NR, Not Reported; ROS, Reactive oxygen species; SCF, Stem cell facto; TGFβ, Transforming growth factor beta; TNFα, Tumor necrosis factor alpha; Treg, T regulatory; VAF, variant allele frequency; VEGF, Vascular endothelial growth factor.

A number of studies has demonstrated that MDS-derived BM-MSCs display defective growth potential (17–19, 21, 24, 30–32, 42, 43). This has been correlated with decreased expression of CD44 and CD49e (32) and at least in some cases it has been associated with increased cellular senescence (18, 19, 33) (**Table 1**). The impaired proliferative capacity of MDS-derived BM-MSCs was corroborated by our study as well (20) and it was suggested that this could be attributed to the decreased expression of the canonical WNT signaling pathway and the concomitant up-regulation of the non-canonical pathway. More recently, Falconi et al., confirmed the down-regulation of the canonical WNT signaling pathway in patient-derived BM-MSCs (40).

As far as the differentiation potential of MDS-derived BM-MSCs is concerned, data are contradictory and these discrepancies might be explained by diversities in patient categories and methodologies across different studies. On one hand, there have been studies demonstrating that these cells do no differ in their capacity to differentiate towards osteoblasts (20, 27, 30, 31, 34), adipocytes (19, 20, 27, 30, 31) and chondrocytes (19, 27, 31) as compared with BM-MSCs derived from healthy donors (**Table 1**). In contrast, reduced osteogenic (19, 21, 24), adipogenic (21) and chondrogenic differentiation (17) have also been reported (**Table 1**). As regards the deregylated osteogenic capacity of BM-MSCs derived from MDS patients, Geyh et al. have demonstrated that it could be partly triggered by TGFβ1 (44). Furthermore, in line with the impaired *in vitro* osteogenic differentiation potential of patients-derived BM-MSCs, an early study on transiliac bone biopsies obtained from MDS patients demonstrated abnormalities in bone remodeling consisting of decreased number of osteoclasts and osteoblasts and decreased bone formation as evidenced by the diminished mineral apposition rate (45). Bone loss in individuals suffering from MDS was also shown in a more recent report (46), which provided evidence that osteoporosis was more prevalent in patients as compared with age-matched controls.

### MDS-derived BM-MSCs exhibit impaired *ex vivo* HSPC supportive capacity

The question whether MDS-derived BM-MSCs can effectively support hematopoiesis has been addressed *in vitro* by co-culturing them with HSPCs. Results are inconsistent and this may due to patient heterogeneity and differences in the experimental protocols in between studies. Within this context, some authors have suggested that patient BM-MSCs are able to sustain the growth of both autologous/leukemic HSPCs (30, 34) and HSPCs derived from healthy individuals (27) (**Table 1**). Muntion et al. (35), (**Table 1**) have shown that exosomes may be involved in the crosstalk between patient-derived BM-MSCs and hematopoietic cells. More precisely *ex-vivo* expanded BM-MSCs from lower risk MDS patients have been demonstrated to secrete exosomes with a different microRNA cargo than their normal counterparts. Patient BM-MSC-derived exosomes are incorporated into CD34^+^ cells from healthy donors and alter their gene expression *via* microRNA transfer (such as miR-10a and miR-15a) and increase their clonogenic potential and viability. Whether BM-MSC derived exosomes similarly support clonal hematopoiesis in the MDS setting remains to be seen. On the other hand, many studies have demonstrated that MDS-derived BM-MSCs have an impaired potential to support normal HSPCs (18–21, 24, 29, 31, 44)(**Table 1**). This could be attributed to TGFβ1 (44) as well as to the defective expression of niche-derived molecules known to be involved in hematopoiesis, such as osteopontin, angiopoietin, jagged-1, kit ligand, hepatocyte growth factor (*HGF*), C-X-C motif chemokine ligand 12 (*CXCL12*), insulin like growth factor-1 (*IGF1)*, insulin growth factor binding protein 2 (*IGFBP2*), thrombopoietin (*TPO*) (18, 19, 29). In addition, in CD271^+^ BM-MSCs from LR-MDS the up-regulation of inflammatory factors and inhibitors of hematopoiesis such as interleukin 6 (*IL-6)*, *IL-8* and C-C motif chemokine ligand *(CCL3) (* 21, 36, 47), the transcription of which is increased secondary to activated NF-kB signaling (36), has been associated with attenuation of HSPC numbers and function *ex vivo.* Finally, CD73^+^ MDS-derived BM-MSCs were shown to have a negative impact on the clonogenic potential of autologous hematopoietic cells, as compared to the effect of CD73^+^ BM-MSCs derived from healthy donors on normal hematopoietic cells (48). This was associated with the increased expression of focal adhesion kinase, a protein involved in various cellular processes including survival, proliferation, differentiation and adhesion (49), in patient-derived BM-MSCs (48).

### Deregulated immunomodulatory functions of MDS-derived BM-MSCs

It has been widely acknowledged that BM-MSCs possess broad immunoregulatory properties, involving cells associated with both innate and adaptive immunity (reviewed in (50)). A large body of evidence supports the role of immune abnormalities in MDS pathogenesis (reviewed in (51)) and accumulating data suggests that BM-MSCs may contribute herein (**Table 1**). More precisely, BM-MSCs from a specific MDS subtype (refractory anemia) deficiently inhibit *in vitro* T cell activation and proliferation (25). Another study has demonstrated that BM-MSCs derived from lower risk MDS patients differ in terms of immunoregulatory properties as compared to those from higher MDS patients (28). More specifically, the former were associated with a lower T cell apoptosis, a less potent inhibitory effect and a lower T-regulatory cell inducible rate. In addition, BM-MSCs from lower risk MDS patients exhibit decreased capacity to inhibit dendritic cell maturation and proliferation as compared to those derived from higher MDS patients (37). Moreover, MDS-derived BM-MSCs, but not those derived from healthy donors, have been reported to induce naive normal monocytes to acquire the properties of myeloid derived suppressor cells and eventually down-regulate NK and T cell function (38). Taken together these findings suggest that MDS-derived BM MSCs have impaired immuneregulatory functions. The fact that in our study (31) BM-MSCs from MDS patients effectively inhibited T cell proliferation might in part be attributed to differences in patient distribution within the MDS subtypes, along with dissimilar experimental protocols as compared to other studies.

### Cytogenetic abnormalities of MDS-derived BM-MSCs

Whereas clonal cytogenetic abnormalities in hematopoietic cells are detected in approximately 40-70% of patients with *de novo* MDS, data regarding the presence of genetic aberrations in MDS-derived BM MSCs have been contradictory. While several studies (17, 27, 52), including ours (53) (**Table 1**) have shown that patient *ex-vivo* expanded BM-MSCs harbor chromosomal aberrations, others report that these cells are normal in terms of cytogenetic analysis (25, 29, 34) (**Table 1**). Interestingly, clonal chromosomal abnormalities detected in MDS-derived BM-MSCs consistently differ from those in hematopoietic cells from the same individual (53), thereby suggesting that patient-derived BM-MSCs and hematopoietic cells do not derive from the same clone. In support of this notion, Fabiani et al. (54) reported that mutations of epigenetic and spliceosomal genes in the BM-mononuclear cells from MDS patients were not present in the mesenchymal compartment.

As some chromosomal aberrations in MDS-derived BM-MSCs were not detected in earlier passages, but only in later passages and as cytogenetic abnormalities were also found in cultured BM-MSCs derived from healthy donors (53), the possibility that these alterations occurred due to *ex-vivo* expansion and/or culture conditions could not be excluded. This issue was clarified in a recent study (33) (**Table 1**) investigating the occurrence of clonal mutations in patient and normal *ex vivo* expanded BM-MSCs by exome sequencing. MDS-derived BM-MSCs were shown to harbor increased mutational burden and distinct mutational signatures as compared to healthy BM-MSCs. However, highly recurrent mutations identified during culture could not be backtracked in primary -non expanded- stroma cells from the same patients (33). These findings suggest that there is no evidence for clonal mutations in the stroma compartment of MDS patients and that the mutations detected during *ex vivo* expansion of MDS-derived BM-MSCs are related to *in vitro* culture *per se*.

### Evidence for deregulations of the BM mesenchymal compartment in MDS mouse models

Conclusive evidence for the key role of the mesenchymal components of the BM microenvironment in the emergence of MDS-like disease in mice was firstly reported by Raaijmakers et al. (55). In this pivotal study, the authors demonstrated that targeted deletion of *Dicer1 -*an RNAse III endonuclease involved in miRNA biogenesis- from murine *osterix-*expressing osteoprogenitors, but not terminally differentiated osteoblasts, resulted in defective osteoblast differentiation and in the initiation and propagation of a form of myelodysplasia associated with leukopenia and lymphocytopenia. Osteolineage cells from mutant mice expressed significantly lower levels of the Schwachman-Bodian-Diamond syndrome (*Sbds*) gene (55). Mutations of this gene are found in Schwachman-Diamond syndrome (SDS) which is characterized by exocrine pancreatic dysfunction, cytopenias- especially neutropenia and bone abnormalities (56). Of note, patients with SDS are at risk for developing MDS and AML (56). S*bds* deletion in murine osteoprogenitors resulted in leukopenia, lymphopenia, and myelodysplasia, thus recapitulating the phenotype of mice harboring *Dicer1* deletion within the same cells (55). Consistent with these findings, reduced expression of *Dicer1* mRNA and protein levels as well down-regulated *Sbds* gene expression were also reported in BM-MSCs from MDS patients (57). On the other hand, a recent study showed that none out of 121 individuals with germline pathogenic *Dicer1* variants developed MDS or leukemia (58). Thus, the clinical significance of Dicer1 in the MDS setting remains elusive. In addition *Sbds* deletion in osterix+ mesenchymal stem/progenitor cells induces genotoxic stress in HSPCs *via* the inflammatory p53-S100A8/9-TLR signaling (59). Activation of this axis seems to have clinical relevance as it was also observed in a subset of lower risk MDS patients who were characterized by a significantly shorter progression free survival and leukemic evolution (59).

The NUP98 gene encodes a protein that is involved in RNA and protein transport across the nuclear membrane (60). *NUP98* fusion genes have been identified in various hematologic malignancies, including MDS. The NUP98-HOXD13 fusion gene has been detected in patients with MDS (60) and mice expressing the hematopoietic compartment specific vav-driven NUP98-HOXD13 fusion transgene eventually develop MDS-like features (46, 60). Interestingly, these mice exhibit also a disrupted microenvironment, which has been reported to contribute significantly in disease progression (46, 61). Alterations in the BM microenvironment include increased number of osteoblasts, reduced numbers of osteoclasts and increased amount of non-mineralized bone (46). Notably, neutralization of the increased serum levels of fibroblast growth factor 23 (FGF23), a regulator of phosphate homeostasis and bone mineralization and an inhibitor of erythropoiesis, restored bone microarchitecture and improved osteoid mineralization and anemia (61). Elevated FGF23 levels and increased amount of non mineralized bone were also observed in samples from MDS patients, thereby corroborating the findings in NHD13 mice (61)

Impaired bone metabolism was also reported recently in the *Abcg2* MDS/AML mouse model (62). In this model, expression of a mutant enhancer of zeste homolog 2 (EZH2-the catalytic subunit of polycomp repressive complex 2, one of the 10 most frequently mutated genes in MDS (63)) lacking the catalytic SET domain (EZH2-dSET) induces Abcg2 (a drug efflux transporter) up-regulation. Mice transplanted with syngeneic bone marrow cells retrovirally tranduced with EZH2-dSET developed an MDS-like disease (63). These mice were also shown to have reduced bone volume, due to decreased bone formation. Similar findings were observed in NHD13 mice and in MDS patients as well. Furthermore, the authors demonstrated that murine MDS/AML cells inhibited the commitment of BM-MSCs towards osteoprogenitors, thereby deregulating the capacity of MSCs to support normal hematopoiesis. This suppression was mediated *via* extracellular vesicles derived from MDS/AML cells (62). Collectively, this data add to the established notion that malignant hematopoietic cells are able to modify MSCs, so that the latter promote disease propagation and evolution.

The *Apc^del/+^* MDS mouse model is a conditional knock-out mouse based on the Mx1-Cre system the most commonly used system to delete the gene of interest in experimental hematology (64–66). Apc is a crucial negative regulator of the canonical b-catenin (Ctnnb1)/WNT-pathway. In the *Apc^del/+^* MDS mouse model the deletion of 1 Apc allele occurs not only in hematopoietic cells but also in BM stromal cells (65, 66). *Apc* haploinsufficiency in the mouse BM niche induces the development of myelodysplasia, which is characterized by severe macrocytic anemia (65). Interestingly, MDS is mediated *via* aberrant WNT signaling in the BM microenvironment (66). In support of this notion drug inhibition of the WNT/Ctnnb1 pathway by the anthelminthic agent pyrvinium delays and/or inhibits MDS development in *Apc* ^del/+^ mice (66).

As far as human MDS is concerned, *in vivo* evidence for the facilitating role of BM-MSCs was provided by a study using a xenograft model (67). More specifically, co-transplantation of CD34^+^ cells derived from lower risk MDS patients along with *ex vivo* expanded BM-MSCs from the same patients in NOD/LtSzcid-IL2rg^-/-^ (NSG) mice significantly increased engraftment of hematopoietic cells as compared to transplantation in the absence of BM-MSCs or with BM-MSCs derived from healthy donors. Furthermore, ex vivo expanded MDS-derived BM-MSCs were shown to differ from their normal counterparts regarding expression of genes related with osteogenesis, adipogenesis, fibrosis, inflammation, cell adhesion, extracellular matrix remodeling and cytokine signaling. Of note, normal MSCs acquired similar properties upon co-culture with patient-derived bone marrow cells, substantiating the issue of hematopoietic MDS cells inducing niche alterations, favoring malignant cell expansion and disease evolution (67). However, in contrast to the aforementioned xenograft model, femoral co-transplantation of BM mononuclear cells derived from MDS patients in combination with autologous or allogeneic patient-derived BM-MSCs or BM-MSCs derived from healthy individuals in NSG mice or in NSG mice with knock-in for human stem cell factor (SCF), granulocyte-macrophage colony-stimulating factor (GM-CSF), and IL-3, did not enhance MDS engraftment (68). The discrepancy between the two studies is not clear. Yet, the fact that in the latter study injected MSCs did not exhibit long-term engraftment suggests that human microenvironment was only transiently established, which might explain -at least in part- the lack of influence of MSCs on MDS engraftment

The contribution of human MDS-derived BM-MSCs for the engraftment and maintenance of MDS HSCs in mice was also corroborated in a recent study by Mian et al. (69). The authors injected patient derived HSCs into gelatin-based scaffolds that were previously seeded with autologous or allogeneic MDS-derived BM-MSCs and the construct was subsequently transplanted in mice. Engraftment of MDS HSCs was achieved in 94% of cases whereas persistent long-term engraftment within scaffolds was observed in 0.2-86%. Interestingly, patient-derived HSCs were shown to move out of the scaffold and home to an adjacent scaffold previously seeded with human BM-MSCs but not with murine BM- MSCs (69), thereby highlighting the critical interplay between bone marrow microenvironment and malignant cells in the MDS setting.

### Therapeutic targeting of BM-MSCs in MDS

The aforementioned preclinical studies support the critical contribution of the BM-MSC compartment in the initiation and/or propagation of MDS and provide the rationale for its therapeutic targeting as a strategy to delay and/or halt disease evolution. In line with this notion, the beneficial effects of some of the therapeutic modalities currently applied in MDS may be exerted *via* amelioration of the BM-MSCs’ impairment, as will be outlined below (**Table 2**).

**Table 2:** Treatment modalities in MDS and their potential effects on patient-derived BM-MSCs.

Pharmaceutical agent	Postulated effects on MDS-derived BM-MSCs
Iron chelation (70, 71)	Restoration of impaired properties ROS down-regulation
Azacitidine (21, 39, 72, 73)	↑ proliferative potential Restoration of aberrant DNA methylation patterns Enhancement of HSPC support Modulation of genes involved in support of hematopoiesis ↓production of HGF and CXCL12 α ↓ adhesion, survival and proliferation of MDS-derived HSPCs
Decitabine (74)	Improvement of impaired properties ↓ proportion of cells in G0/G1 phase ↓ expression of *CDKN1A* ↓ differentiation of T cells to Tregs
Lenalidomide (18)	↓ CXCL12 secretion and ↑ of dormant MDS-derived HSPCs from their niches Enhancement of normal HSPCs’ support
Luspatercept (75)	↑ secretion of CXCL12 ↓ SMAD 2/3 activation ↑ adherence of HSPCs ↑ clonogenic potential of HSPCs

BM-MSCs, Bone Marrow Mesenchymal Stromal Cells; CAFCs, cobblestone-area forming cells; CDKN1A cyclin dependent kinase inhibitor 1A; CXCL12, C-X-C motif chemokine ligand 12; HGF, Hepatocyte growth factor; HSPCs, Hematopoietic stem/progenitor cells; MDS, Myelodysplastic Syndrome; ROS, Reactive oxygen species; SCF, Stem cell factor; TGFβ, Transforming growth factor beta; TNFα, Tumor necrosis factor alpha; Treg cells, T regulatory cells; ↑ means increase, ↓ means decrease.

Iron overload (IO) is a common finding in MDS patients that occurs as a result of ineffective hematopoiesis as well as red blood cell transfusions (76, 77). Aside from its deleterious effect on hematopoiesis (76, 77) IO has also been demonstrated to impair BM-MSCs. More precisely, Huang et al. (78) have demonstrated that BM-MSCs from iron overloaded higher-risk MDS patients exhibit decreased quantity, defective proliferation capacity and reduced osteogenic differentiation potential. Furthermore, they express lower levels of hematopoiesis-associated genes such as *VEGFA, CXCL12*, and *TGFβ1 (* 78). In addition IO induces apoptosis in MDS-derived BM-MSCs *via* increased level of reactive oxygen species (ROS) and the ROS-associated Wnt/b-catenin pathway (78). IO has also been reported to induce mitochondrial fragmentation *via* ROS and the activation of the AMPK/MFF/Drp1 pathway in MDS-derived BM-MSCs. Of note, all these effects of IO in patient BM-MSCs are reversed, at least in part, by antioxidants and iron chelation. Moreover, in an IO mouse model, antioxidant and iron chelation have been shown to partially restore the defective BM-MSCs’ hematopoietic support. Iron chelation is commonly administered in MDS patients, especially in those with lower-risk MDS and its treatment is associated with improvement of cytopenias in a significant proportion of patients (70). The aforementioned data imply that in the MDS setting the beneficial role of iron chelation in hematopoiesis may also result from the restoration, to a certain extent, of the MSCs deficits and further support the application of this treatment in myelodysplasia.

On the other hand Hu et al. (71) have reported that IO upregulates the expression of IL-6, IL-8, TGFβ and VEGF in MDS-derived BM-MSCs through ROS upregulation and subsequent HIF-1a overexpression (71). Antioxidants and iron chelation down-regulated the levels of the aforementioned cytokines. These data provide the theoretical background for probing more deeply into the role of HIF-1a in the BM microenvironment in MDS and provide further justification for the use of HIF-1 inhibitors in this disorder, an issue that is currently under investigation (79). Of note, the discrepancies in terms of the effect of IO in the expression of VEGF and TGFβ between the study Huang et al. (56) and that of Hu et al. (71) may reflect differences in patient cohorts regarding disease categorization and IPSS risk classification.

Previous studies have demonstrated that *ex vivo* expanded MDS-derived BM-MSCs have significantly different DNA methylation patterns from their normal counterparts (19, 21)and are characterized by aberrant hypermethylation (19, 39). These findings set the stage for the investigation of the potential effect on patient MSCs of the hypomethylating agent azacitidine (AZA), which is widely used in higher risk MDS and may also be effective in lower risk MDS refractory to first line treatments (80, 81). AZA treatment has thus been shown to restore the aberrant hypermethylation pattern of MDS-derived BM-MSCs (39) to increase their proliferation potential and osteogenic capacity, and improve their ability to support HSPCs for *in vivo* engraftment (21). Another study (72) has reported that in patient BM-MSCs AZA regulates various genes involved in the support of hematopoiesis, essentially genes associated with IFN-γ and extracellular matrix receptor interaction pathways. This transcriptional modulation could account for the improved capacity of AZA-treated MDS-derived BM-MSCs to preferentially support healthy-derived HSPCs, over patient-derived HSPCs (72). AZA has also been shown to upregulate the expression of the serine protease inhibitor kunitz-type2 (SPINT2/HAI-2), an inhibitor of hepatocyte growth factor (HGF) activation, in patient-derived BM-MSCs (73). Based on data derived from the HS-5 stromal line, it has been hypothesized that AZA may down-regulate HGF and CXL12 production by MDS-MSCs and consequently increase cell-adhesion, proliferation and survival of MDS-derived HSPCs (73). The aforementioned findings provide evidence for the potential of AZA in targeting the defective BM-MSCs, thereby extending the effects of this therapeutic modality in the MDS-setting.

Decitabine is another hypomethylating agent which has been approved by the Food and Drug Administration (FDA) for the treatment of patients with MDS (82). Incubation of culture expanded BM-MSCs from MDS patients in the presence of Decitabine resulted in a significant decrease in the proportion of cells in the G0/G1 phases as compared to MDS-derived BM-MSCs incubated in the absence of the drug (control group). This effect was associated with a reduced gene expression of cyclin dependent kinase inhibitor 1A *(CDKN1A) (* 74). Moreover the ability of BM-MSCs from MDS patients incubated in the presence of decitabine to induce the differentiation of T cells into Tregs was significantly reduced compared with control BM-MSCs and this was linked to decreased gene expression of programmed death-ligand 1 (*PDL1*) (74). Taken together these findings suggest that decitabine may improve, at least to a certain extent, the impaired properties of MDS-derived BM-MSCs

Lenalidomide is an immunomodulatory agent showing efficacy in lower risk MDS (80, 81). Of note, apart from acting directly on MDS cells, lenalidomide has been demonstrated to target the BM microenvironment as well (83), including BM-MSCs (18). More precisely lenalidomide reduces CXCL12 secretion by MDS-derived BM-MSCs and this has been hypothesized to induce the egress of dormant MDS cells from their niches and render them more sensitive to the drug. In addition, treatment of *ex vivo* expanded lower risk MDS-derived BM-MSCs with lenalidomide has been reported to improve their capacity to support normal clonogenic HSPCs (18).

The downstream key mediators of TGF-β superfamily signaling, SMAD2/3 are constitutively activated in MDS CD34^+^ cells and this has been associated with impaired late-stage erythroid maturation and subsequent anemia (84, 85). Luspatercept is a therapeutic agent that sequesters TGF-β superfamily ligands and consequently decreases SMAD2/3 activation thereby restoring erythroid maturation and improving anemia. It has been approved for the treatment of selected patient with lower risk MDS (84, 85). Interestingly, a recent study has provided evidence that luspatercept affects also the BM-MSCs (75). More precisely, it decreases SMAD2/3 activation in ex-vivo expanded MDS-derived BM-MSCs and increases CXCL12 secretion. Furthermore, pre-treatment of MDS-derived BM-MSCs with luspatercept improved the clonogenic potential of co-cultured HSPCs, their adherence and expression of CXCR4, the CXCL12 receptor, as well as their homing in zebrafish embryos. Finally, BM-MSCs derived from patients treated with luspatercept exhibited a greater capacity to sustain the clonogenic potential of normal, but not MDS-derived HSPCs (75).

As previously mentioned, MDS-derived hematopoietic cells have been demonstrated to impair BM-MSCs (62, 67). Therefore, it is tempting to speculate that the therapeutic effect of allogeneic HSPC transplantation, which remains the only curative option in MDS (80), may also be exerted *via* favorable modulations of the functions and properties of the mesenchymal compartment. This issue requires further investigation by comparative analyses on the characteristics of MDS-derived BM-MSCs prior and post allogeneic BM transplantation. To the best of our knowledge, such studies have not been reported thus far.

## Conclusions

We have summarized herein the current views on the implication of BM-MSCs in the MDS pathogenesis. Collectively, there is large number of studies that support the notion that BM-MSCs play a major part in the hematopoietic failure that characterizes MDS and demonstrate their involvement in the initiation and progression of the disease. The interactions between BM-MSCs and HSCs have been historically assessed *in vitro*. Although discrepancies exist, due to different experimental approaches and patient heterogeneity in between studies, most reports have shown aberrancies in patient-derived BM-MSCs and defective hematopoiesis-supporting capacity. Nevertheless, *in vitro* findings may not adequately recapitulate the complexity of the BM and thus may not allow for the accurate dissection of the interactions between BM-MSCs and HSPCs within the niche. More recently, mouse models of MDS and xenotransplantation studies have shed some light into the crosstalk between BM-MSCs and their progeny on one hand and HSPCs on the other.Evidence has been provided for two –non mutually exclusive – aspects of the role of BM-MSCs in MDS pathophysiology: a) aberrant BM-MSCs promote the development of the myeloid malignancy and b) malignant HSPCs alter BM-MSCs causing them to facilitate disease propagation and/or evolution. Based on these notions a matter of utmost importance that has not been investigated thus far is the delineation of the potential impact of BM-MSCs in the setting of clonal hematopoiesis detected in individuals with otherwise normal complete blood cell counts and without any overt underlying hematological malignancy (86). Such clonal hematopoiesis, called clonal hematopoiesis of indeterminate potential (CHIP) (86) may be linked to aging (87) and is characterized by mutations in genes associated with MDS and AML (86, 87). Notably, CHIP has been associated with an increased risk of hematologic malignancies (87, 88), including MDS. Whether perturbed BM-MSCs can create a permissive soil herein and thus contribute to malignant transformation of CHIP cases remains to be seen.

## Future perspectives

At present, we have only gained a first insight on the dependence of MDS cells on the BM-microenvironment and lots of issues still remain obscure. The emergence of studies using humanized bone marrow like structures (89, 90) that employ the seeding of AML/MDS-derived BM-MSCs on appropriate scaffolds, the subsequent implantation of AML/MDS-HSPCs into the scaffolds and finally the introduction of the constructs subcutaneously into a mouse is anticipated to provide a robust and accurate mode that will pave the way for the decoding the complex interplay between AML/MDS cells and their microenvironment. In addition, as the critical contribution of BM-MSCs in MDS pathophysiology is being acknowledged, there is an increasing interest in their potential therapeutic targeting. Of note, some of the already available MDS treatments have indeed been recognized to affect the mesenchymal compartment as well. However, as the interactions between HSPCs and BM-MSCs in the MDS setting is progressively being unraveled, opportunities for the development of novel therapies to regulate this crosstalk is expected to emerge and possibly change the treatment landscape in this myeloid malignancy.

## Author contributions

Conceptualization, Writing-Review and Editing, CP. Writing-Original Draft, AM. Writing-Review and Editing, HP. All authors contributed to the article and approved the submitted version.

## References

[1] Comazzetto S Shen B Morrison SJ. Niches that regulate stem cells and hematopoiesis in adult bone marrow. Dev Cell (2021) 56(13):1848–60. doi: 10.1016/j.devcel.2021.05.018 PMC8282762 34146467

[2] Hoggatt J Kfoury Y Scadden DT. Hematopoietic stem cell niche in health and disease. Annu Rev Pathol (2016) 11:555–81. doi: 10.1146/annurev-pathol-012615-044414 27193455

[3] Pinho S Frenette PS. Haematopoietic stem cell activity and interactions with the niche. Nat Rev Mol Cell Biol (2019) 20(5):303–20. doi: 10.1038/s41580-019-0103-9 PMC6483843 30745579

[4] Xiao Y McGuinness CS Doherty-Boyd WS Salmeron-Sanchez M Donnelly H Dalby MJ. Current insights into the bone marrow niche: From biology in vivo to bioengineering ex vivo. Biomaterials (2022) 286:121568. doi: 10.1016/j.biomaterials.2022.121568 35580474

[5] Pontikoglou C Deschaseaux F Sensebe L Papadaki HA. Bone marrow mesenchymal stem cells: Biological properties and their role in hematopoiesis and hematopoietic stem cell transplantation. Stem Cell Rev (2011) 7(3):569–89. doi: 10.1007/s12015-011-9228-8 21249477

[6] Ding L Saunders TL Enikolopov G Morrison SJ. Endothelial and perivascular cells maintain haematopoietic stem cells. Nature (2012) 481(7382):457–62. doi: 10.1038/nature10783 PMC3270376 22281595

[7] Sugiyama T Kohara H Noda M Nagasawa T. Maintenance of the hematopoietic stem cell pool by Cxcl12-Cxcr4 chemokine signaling in bone marrow stromal cell niches. Immunity (2006) 25(6):977–88. doi: 10.1016/j.immuni.2006.10.016 17174120

[8] Zhang J Niu C Ye L Huang H He X Tong WG. Identification of the haematopoietic stem cell niche and control of the niche size. Nature (2003) 425(6960):836–41. doi: 10.1038/nature02041 14574412

[9] Kunisaki Y Bruns I Scheiermann C Ahmed J Pinho S Zhang D. Arteriolar niches maintain haematopoietic stem cell quiescence. Nature (2013) 502(7473):637–43. doi: 10.1038/nature12612 PMC3821873 24107994

[10] Acar M Kocherlakota KS Murphy MM Peyer JG Oguro H Inra CN. Deep imaging of bone marrow shows non-dividing stem cells are mainly perisinusoidal. Nature (2015) 526(7571):126–30. doi: 10.1038/nature15250 PMC4850557 26416744

[11] Dominici M Le BK Mueller I Slaper-Cortenbach I Marini F Krause D. Minimal criteria for defining multipotent mesenchymal stromal cells. the international society for cellular therapy position statement. Cytotherapy (2006) 8(4):315–7. doi: 10.1080/14653240600855905 16923606

[12] Crippa S Bernardo ME. Mesenchymal stromal cells: Role in the bm niche and in the support of hematopoietic stem cell transplantation. HemaSphere (2018) 2(6):e151. doi: 10.1097/hs9.0000000000000151 31723790 PMC6745957

[13] Crippa S Santi L Berti M De Ponti G Bernardo ME. Role of ex vivo expanded mesenchymal stromal cells in determining hematopoietic stem cell transplantation outcome. Front Cell Dev Biol (2021) 9:663316. doi: 10.3389/fcell.2021.663316 34017834 PMC8129582

[14] Adès L Itzykson R Fenaux P. Myelodysplastic syndromes. Lancet (London England) (2014) 383(9936):2239–52. doi: 10.1016/s0140-6736(13)61901-7 24656536

[15] Raaijmakers MH. Myelodysplastic syndromes: Revisiting the role of the bone marrow microenvironment in disease pathogenesis. IntJHematol (2012) 95(1):17–25. doi: 10.1007/s12185-011-1001-x 22218882

[16] Kastrinaki MC Pontikoglou C Klaus M Stavroulaki E Pavlaki K Papadaki HA. Biologic characteristics of bone marrow mesenchymal stem cells in myelodysplastic syndromes. Curr Stem Cell Res Ther (2011) 6(2):122–30. doi: 10.2174/157488811795495422 20528751

[17] Lopez-Villar O Garcia JL Sanchez-Guijo FM Robledo C Villaron EM Hernández-Campo P. Both expanded and uncultured mesenchymal stem cells from mds patients are genomically abnormal, showing a specific genetic profile for the 5q- syndrome. Leukemia (2009) 23(4):664–72. doi: 10.1038/leu.2008.361 19151777

[18] Ferrer RA Wobus M List C Wehner R Schönefeldt C Brocard B. Mesenchymal stromal cells from patients with myelodyplastic syndrome display distinct functional alterations that are modulated by lenalidomide. Haematologica (2013) 98(11):1677–85. doi: 10.3324/haematol.2013.083972 PMC3815166 23716561

[19] Geyh S Oz S Cadeddu RP Frobel J Bruckner B Kundgen A. Insufficient stromal support in mds results from molecular and functional deficits of mesenchymal stromal cells. Leukemia (2013) 27(9):1841–51. doi: 10.1038/leu.2013.193 23797473

[20] Pavlaki K Pontikoglou CG Demetriadou A Batsali AK Damianaki A Simantirakis E. Impaired proliferative potential of bone marrow mesenchymal stromal cells in patients with myelodysplastic syndromes is associated with abnormal wnt signaling pathway. Stem Cells Dev (2014) 23(14):1568–81. doi: 10.1089/scd.2013.0283 24617415

[21] Poon Z Dighe N Venkatesan SS Cheung AMS Fan X Bari S. Bone marrow mscs in mds: Contribution towards dysfunctional hematopoiesis and potential targets for disease response to hypomethylating therapy. Leukemia (2019) 33(6):1487–500. doi: 10.1038/s41375-018-0310-y PMC6756222 30575819

[22] Weickert MT Hecker JS Buck MC Schreck C Rivière J Schiemann M. Bone marrow stromal cells from mds and aml patients show increased adipogenic potential with reduced delta-Like-1 expression. Sci Rep (2021) 11(1):5944. doi: 10.1038/s41598-021-85122-8 33723276 PMC7961144

[23] Flores-Figueroa E Arana-Trejo RM Gutiérrez-Espíndola G Pérez-Cabrera A Mayani H. Mesenchymal stem cells in myelodysplastic syndromes: Phenotypic and cytogenetic characterization. Leuk Res (2005) 29(2):215–24. doi: 10.1016/j.leukres.2004.06.011 15607371

[24] Varga G Kiss J Várkonyi J Vas V Farkas P Pálóczi K. Inappropriate notch activity and limited mesenchymal stem cell plasticity in the bone marrow of patients with myelodysplastic syndromes. Pathol Oncol Res POR (2007) 13(4):311–9. doi: 10.1007/bf02940310 18158566

[25] Han Q Sun Z Liu L Chen B Cao Y Li K. Impairment in immuno-modulatory function of Flk1(+)Cd31(-)Cd34(-) mscs from mds-Ra patients. Leuk Res (2007) 31(11):1469–78. doi: 10.1016/j.leukres.2006.12.016 17360037

[26] Zhi-Gang Z Wei-Ming L Zhi-Chao C Yong Y Ping Z. Immunosuppressive properties of mesenchymal stem cells derived from bone marrow of patient with hematological malignant diseases. Leuk Lymphoma (2008) 49(11):2187–95. doi: 10.1080/10428190802455875 19021063

[27] Flores-Figueroa E Montesinos JJ Flores-Guzman P Gutierrez-Espindola G Arana-Trejo RM Castillo-Medina S. Functional analysis of myelodysplastic syndromes-derived mesenchymal stem cells. Leuk Res (2008) 32(9):1407–16. doi: 10.1016/j.leukres.2008.02.013 18405968

[28] Zhao Z Wang Z Li Q Li W You Y Zou P. The different immunoregulatory functions of mesenchymal stem cells in patients with low-risk or high-risk myelodysplastic syndromes. PloS One (2012) 7(9):e45675. doi: 10.1371/journal.pone.0045675 23029178 PMC3448671

[29] Zhao ZG Xu W Yu HP Fang BL Wu SH Li F. Functional characteristics of mesenchymal stem cells derived from bone marrow of patients with myelodysplastic syndromes. Cancer Lett (2012) 317(2):136–43. doi: 10.1016/j.canlet.2011.08.030 22240014

[30] Corradi G Baldazzi C Očadlíková D Marconi G Parisi S Testoni N. Mesenchymal stromal cells from myelodysplastic and acute myeloid leukemia patients display in vitro reduced proliferative potential and similar capacity to support leukemia cell survival. Stem Cell Res Ther (2018) 9(1):271. doi: 10.1186/s13287-018-1013-z 30359303 PMC6202844

[31] Klaus M Stavroulaki E Kastrinaki MC Fragioudaki P Giannikou K Psyllaki M. Reserves, functional, immunoregulatory, and cytogenetic properties of bone marrow mesenchymal stem cells in patients with myelodysplastic syndromes. Stem Cells Dev (2010) 19(7):1043–54. doi: 10.1089/scd.2009.0286 19788374

[32] Aanei CM Flandrin P Eloae FZ Carasevici E Guyotat D Wattel E. Intrinsic growth deficiencies of mesenchymal stromal cells in myelodysplastic syndromes. Stem Cells Dev (2012) 21(10):1604–15. doi: 10.1089/scd.2011.0390 PMC3376465 21933023

[33] Jann J-C Mossner M Riabov V Altrock E Schmitt N Flach J. Bone marrow derived stromal cells from myelodysplastic syndromes are altered but not clonally mutated in vivo. Nat Commun (2021) 12(1):6170. doi: 10.1038/s41467-021-26424-3 34697318 PMC8546146

[34] Soenen-Cornu V Tourino C Bonnet ML Guillier M Flamant S Kotb R. Mesenchymal cells generated from patients with myelodysplastic syndromes are devoid of chromosomal clonal markers and support short- and long-term hematopoiesis in vitro. Oncogene (2005) 24(15):2441–8. doi: 10.1038/sj.onc.1208405 15735749

[35] Muntión S Ramos TL Diez-Campelo M Rosón B Sánchez-Abarca LI Misiewicz-Krzeminska I. Microvesicles from mesenchymal stromal cells are involved in hpc-microenvironment crosstalk in myelodysplastic patients. PloS One (2016) 11(2):e0146722. doi: 10.1371/journal.pone.0146722 26836120 PMC4737489

[36] Ping Z Chen S Hermans SJF Kenswil KJG Feyen J van Dijk C. Activation of nf-Kb driven inflammatory programs in mesenchymal elements attenuates hematopoiesis in low-risk myelodysplastic syndromes. Leukemia (2019) 33(2):536–41. doi: 10.1038/s41375-018-0267-x PMC6365382 30315231

[37] Wang Z Tang X Xu W Cao Z Sun L Li W. The different immunoregulatory functions on dendritic cells between mesenchymal stem cells derived from bone marrow of patients with low-risk or high-risk myelodysplastic syndromes. PloS One (2013) 8(3):e57470. doi: 10.1371/journal.pone.0057470 23469196 PMC3587596

[38] Sarhan D Wang J Sunil Arvindam U Hallstrom C Verneris MR Grzywacz B. Mesenchymal stromal cells shape the mds microenvironment by inducing suppressive monocytes that dampen nk cell function. JCI Insight (2020) 5(5):e130155. doi: 10.1172/jci.insight.130155 PMC7141401 32045384

[39] Bhagat TD Chen S Bartenstein M Barlowe AT Von Ahrens D Choudhary GS. Epigenetically aberrant stroma in mds propagates disease *Via* Wnt/Beta-catenin activation. Cancer Res (2017) 77(18):4846–57. doi: 10.1158/0008-5472.CAN-17-0282 PMC5600853 28684528

[40] Falconi G Fabiani E Fianchi L Criscuolo M Raffaelli CS Bellesi S. Impairment of Pi3k/Akt and Wnt/B-catenin pathways in bone marrow mesenchymal stem cells isolated from patients with myelodysplastic syndromes. Exp Hematol (2016) 44(1):75–83.e1-4. doi: 10.1016/j.exphem.2015.10.005 26521017

[41] Campioni D Rizzo R Stignani M Melchiorri L Ferrari L Moretti S. A decreased positivity for Cd90 on human mesenchymal stromal cells (Mscs) is associated with a loss of immunosuppressive activity by mscs. Cytom Part B Clin Cytom (2009) 76(3):225–30. doi: 10.1002/cyto.b.20461 18985728

[42] Wu Y Aanei CM Kesr S Picot T Guyotat D Campos Catafal L. Impaired expression of focal adhesion kinase in mesenchymal stromal cells from low-risk myelodysplastic syndrome patients. Front Oncol (2017) 7:164. doi: 10.3389/fonc.2017.00164 28848706 PMC5551509

[43] Aanei CM Catafal LC. Evaluation of bone marrow microenvironment could change how myelodysplastic syndromes are diagnosed and treated. Cytom Part A (2018) 93(9):916–28. doi: 10.1002/cyto.a.23506 30211968

[44] Geyh S Rodríguez-Paredes M Jäger P Koch A Bormann F Gutekunst J. Transforming growth factor B1-mediated functional inhibition of mesenchymal stromal cells in myelodysplastic syndromes and acute myeloid leukemia. Haematologica (2018) 103(9):1462–71. doi: 10.3324/haematol.2017.186734 PMC6119130 29773599

[45] Mellibovsky L Diez A Serrano S Aubia J Pérez-Vila E Mariñoso ML. Bone remodeling alterations in myelodysplastic syndrome. Bone (1996) 19(4):401–5. doi: 10.1016/s8756-3282(96)00210-4 8894147

[46] Weidner H Rauner M Trautmann F Schmitt J Balaian E Mies A. Myelodysplastic syndromes and bone loss in mice and men. Leukemia (2017) 31(4):1003–7. doi: 10.1038/leu.2017.7 28074069

[47] Chen S Zambetti NA Bindels EM Kenswill K Mylona AM Adisty NM. Massive parallel rna sequencing of highly purified mesenchymal elements in low-risk mds reveals tissue-Context-Dependent activation of inflammatory programs. Leukemia (2016) 30(9):1938–42. doi: 10.1038/leu.2016.91 PMC5240018 27109510

[48] Aanei CM Eloae FZ Flandrin-Gresta P Tavernier E Carasevici E Guyotat D. Focal adhesion protein abnormalities in myelodysplastic mesenchymal stromal cells. Exp Cell Res (2011) 317(18):2616–29. doi: 10.1016/j.yexcr.2011.08.007 21871449

[49] Schaller MD. Cellular functions of fak kinases: Insight into molecular mechanisms and novel functions. J Cell Sci (2010) 123(Pt 7):1007–13. doi: 10.1242/jcs.045112 20332118

[50] Matheakakis A Batsali A Papadaki HA Pontikoglou CG. Therapeutic implications of mesenchymal stromal cells and their extracellular vesicles in autoimmune diseases: From biology to clinical applications. Int J Mol Sci (2021) 22(18):10132. doi: 10.3390/ijms221810132 PMC8468750 34576296

[51] Peng X Zhu X Di T Tang F Guo X Liu Y. The yin-yang of immunity: Immune dysregulation in myelodysplastic syndrome with different risk stratification. Front Immunol (2022) 13:994053. doi: 10.3389/fimmu.2022.994053 36211357 PMC9537682

[52] Blau O Baldus CD Hofmann WK Thiel G Nolte F Burmeister T. Mesenchymal stromal cells of myelodysplastic syndrome and acute myeloid leukemia patients have distinct genetic abnormalities compared with leukemic blasts. Blood (2011) 118(20):5583–92. doi: 10.1182/blood-2011-03-343467 PMC3217359 21948175

[53] Kouvidi E Stratigi A Batsali A Mavroudi I Mastrodemou S Ximeri M. Cytogenetic evaluation of mesenchymal Stem/Stromal cells from patients with myelodysplastic syndromes at different time-points during ex vivo expansion. Leuk Res (2016) 43:24–32. doi: 10.1016/j.leukres.2016.02.007 26930455

[54] Fabiani E Falconi G Fianchi L Guidi F Bellesi S Voso MT. Mutational analysis of bone marrow mesenchymal stromal cells in myeloid malignancies. Exp Hematol (2014) 42(9):731–3. doi: 10.1016/j.exphem.2014.04.011 24796317

[55] Raaijmakers MH Mukherjee S Guo S Zhang S Kobayashi T Schoonmaker JA. Bone progenitor dysfunction induces myelodysplasia and secondary leukaemia. Nature (2010) 464(7290):852–7. doi: 10.1038/nature08851 PMC3422863 20305640

[56] Huang JN Shimamura A. Clinical spectrum and molecular pathophysiology of shwachman-diamond syndrome. Curr Opin Hematol (2011) 18(1):30–5. doi: 10.1097/MOH.0b013e32834114a5 PMC3485416 21124213

[57] Santamaria C Muntion S Roson B Blanco B Lopez-Villar O Carrancio S. Impaired expression of dicer, drosha, sbds and some micrornas in mesenchymal stromal cells from myelodysplastic syndrome patients. Haematologica (2012) 97(8):1218–24. doi: 10.3324/haematol.2011.054437 PMC3409820 22371183

[58] Vasta LM Khan NE Higgs CP Harney LA Carr AG Harris AK. Hematologic indices in individuals with pathogenic germline Dicer1 variants. Blood Adv (2021) 5(1):216–23. doi: 10.1182/bloodadvances.2020002651 PMC7805337 33570641

[59] Zambetti NA Ping Z Chen S Kenswil KJG Mylona MA Sanders MA. Mesenchymal inflammation drives genotoxic stress in hematopoietic stem cells and predicts disease evolution in human pre-leukemia. Cell Stem Cell (2016) 19(5):613–27. doi: 10.1016/j.stem.2016.08.021 27666011

[60] Lin YW Slape C Zhang Z Aplan PD. Nup98-Hoxd13 transgenic mice develop a highly penetrant, severe myelodysplastic syndrome that progresses to acute leukemia. Blood (2005) 106(1):287–95. doi: 10.1182/blood-2004-12-4794 PMC1201424 15755899

[61] Weidner H Baschant U Lademann F Ledesma Colunga MG Balaian E Hofbauer C. Increased fgf-23 levels are linked to ineffective erythropoiesis and impaired bone mineralization in myelodysplastic syndromes. JCI Insight (2020) 5(15):e137062. doi: 10.1172/jci.insight.137062 PMC7455070 32759495

[62] Hayashi Y Kawabata KC Tanaka Y Uehara Y Mabuchi Y Murakami K. Mds cells impair osteolineage differentiation of mscs *Via* extracellular vesicles to suppress normal hematopoiesis. Cell Rep (2022) 39(6):110805. doi: 10.1016/j.celrep.2022.110805 35545056

[63] Kawabata KC Hayashi Y Inoue D Meguro H Sakurai H Fukuyama T. High expression of Abcg2 induced by Ezh2 disruption has pivotal roles in mds pathogenesis. Leukemia (2018) 32(2):419–28. doi: 10.1038/leu.2017.227 28720764

[64] Velasco-Hernandez T Säwén P Bryder D Cammenga J. Potential pitfalls of the Mx1-cre system: Implications for experimental modeling of normal and malignant hematopoiesis. Stem Cell Rep (2016) 7(1):11–8. doi: 10.1016/j.stemcr.2016.06.002 PMC4945592 27373927

[65] Stoddart A Wang J Fernald AA Karrison T Anastasi J Le Beau MM. Cell intrinsic and extrinsic factors synergize in mice with haploinsufficiency for Tp53, and two human Del(5q) genes, Egr1 and apc. Blood (2014) 123(2):228–38. doi: 10.1182/blood-2013-05-506568 PMC3888289 24264229

[66] Stoddart A Wang J Hu C Fernald AA Davis EM Cheng JX. Inhibition of wnt signaling in the bone marrow niche prevents the development of mds in the Apc(Del/+) mds mouse model. Blood (2017) 129(22):2959–70. doi: 10.1182/blood-2016-08-736454 PMC5454335 28348148

[67] Medyouf H Mossner M Jann JC Nolte F Raffel S Herrmann C. Myelodysplastic cells in patients reprogram mesenchymal stromal cells to establish a transplantable stem cell niche disease unit. Cell Stem Cell (2014) 14(6):824–37. doi: 10.1016/j.stem.2014.02.014 24704494

[68] Krevvata M Shan X Zhou C Dos Santos C Habineza Ndikuyeze G Secreto A. Cytokines increase engraftment of human acute myeloid leukemia cells in immunocompromised mice but not engraftment of human myelodysplastic syndrome cells. Haematologica (2018) 103(6):959–71. doi: 10.3324/haematol.2017.183202 PMC6058784 29545344

[69] Mian SA Bonnet D. Nature or nurture? role of the bone marrow microenvironment in the genesis and maintenance of myelodysplastic syndromes. Cancers (Basel) (2021) 13(16):4116. doi: 10.3390/cancers13164116 PMC8394536 34439269

[70] Parisi S Finelli C. Prognostic factors and clinical considerations for iron chelation therapy in myelodysplastic syndrome patients. J Blood Med (2021) 12:1019–30. doi: 10.2147/jbm.s287876 PMC8651046 34887690

[71] Hu J Meng F Hu X Huang L Liu H Liu Z. Iron overload regulate the cytokine of mesenchymal stromal cells through Ros/Hif-1α pathway in myelodysplastic syndromes. Leuk Res (2020) 93:106354. doi: 10.1016/j.leukres.2020.106354 32380365

[72] Wenk C Garz AK Grath S Huberle C Witham D Weickert M. Direct modulation of the bone marrow mesenchymal stromal cell compartment by azacitidine enhances healthy hematopoiesis. Blood Adv (2018) 2(23):3447–61. doi: 10.1182/bloodadvances.2018022053 PMC6290099 30518537

[73] Roversi FM Cury NM Lopes MR Ferro KP Machado-Neto JA Alvarez MC. Up-regulation of Spint2/Hai-2 by azacytidine in bone marrow mesenchymal stromal cells affects leukemic stem cell survival and adhesion. J Cell Mol Med (2019) 23(2):1562–71. doi: 10.1111/jcmm.14066 PMC6349149 30484958

[74] Pang Y Geng S Zhang H Lai P Liao P Zeng L. Phenotype of mesenchymal stem cells from patients with myelodyplastic syndrome maybe partly modulated by decitabine. Oncol Lett (2019) 18(5):4457–66. doi: 10.3892/ol.2019.10788 PMC6781515 31611955

[75] Wobus M Mies A Asokan N Oelschlägel U Möbus K Winter S. Luspatercept restores sdf-1-Mediated hematopoietic support by mds-derived mesenchymal stromal cells. Leukemia (2021) 35 (10):2936–2947. doi: 10.1038/s41375-021-01275-5 PMC8478655 34002031

[76] Gattermann N. Iron overload in myelodysplastic syndromes (Mds). Int J Hematol (2018) 107(1):55–63. doi: 10.1007/s12185-017-2367-1 29177643

[77] Brissot E Bernard DG Loréal O Brissot P Troadec M-B. Too much iron: A masked foe for leukemias. Blood Rev (2020) 39:100617. doi: 10.1016/j.blre.2019.100617 31753415

[78] Huang L Liu Z. Iron overload impairs bone marrow mesenchymal stromal cells from higher-risk mds patients by regulating the ros-related Wnt/B-catenin pathway. Stem Cells Int (2020) 2020:8855038. doi: 10.1155/2020/8855038 33178287 PMC7648692

[79] Henry DH Glaspy J. Roxadustat for the treatment of anemia in patients with lower-risk myelodysplastic syndrome: Open-label, dose-selection, lead-in stage of a phase 3 study. Am J Hematol (2022) 97(2):174–84. doi: 10.1002/ajh.26397 34724251

[80] Fenaux P Haase D Santini V Sanz GF Platzbecker U Mey U. Myelodysplastic syndromes: Esmo clinical practice guidelines for diagnosis, treatment and follow-up(†☆). Ann Oncol (2021) 32(2):142–56. doi: 10.1016/j.annonc.2020.11.002 33221366

[81] Carraway HE Saygin C. Therapy for lower-risk mds. Hematol Am Soc Hematol Educ Program (2020) 2020(1):426–33. doi: 10.1182/hematology.2020000127 PMC7727572 33275714

[82] Short NJ Kantarjian H. Hypomethylating agents for the treatment of myelodysplastic syndromes and acute myeloid leukemia: Past discoveries and future directions. Am J Hematol (2022) 97(12):1616–1626. doi: 10.1002/ajh.26667 35871436

[83] Stahl M Zeidan AM. Lenalidomide use in myelodysplastic syndromes: Insights into the biologic mechanisms and clinical applications. Cancer (2017) 123(10):1703–13. doi: 10.1002/cncr.30585 28192601

[84] Verma A Suragani RN Aluri S Shah N Bhagat TD Alexander MJ. Biological basis for efficacy of activin receptor ligand traps in myelodysplastic syndromes. J Clin Invest (2020) 130(2):582–9. doi: 10.1172/jci133678 PMC6994157 31961337

[85] Kubasch AS Fenaux P Platzbecker U. Development of luspatercept to treat ineffective erythropoiesis. Blood Adv (2021) 5(5):1565–75. doi: 10.1182/bloodadvances.2020002177 PMC7948289 33687432

[86] Steensma DP Bejar R Jaiswal S Lindsley RC Sekeres MA Hasserjian RP. Clonal hematopoiesis of indeterminate potential and its distinction from myelodysplastic syndromes. Blood (2015) 126(1):9–16. doi: 10.1182/blood-2015-03-631747 25931582 PMC4624443

[87] Gondek LP. Chip: Is clonal hematopoiesis a surrogate for aging and other disease? Hematol Am Soc Hematol Educ Program (2021) 2021(1):384–9. doi: 10.1182/hematology.2021000270 PMC8791098 34889429

[88] Jaiswal S Fontanillas P Flannick J Manning A Grauman PV Mar BG. Age-related clonal hematopoiesis associated with adverse outcomes. New Engl J Med (2014) 371(26):2488–98. doi: 10.1056/NEJMoa1408617 PMC4306669 25426837

[89] Antonelli A Noort WA Jaques J de Boer B de Jong-Korlaar R Brouwers-Vos AZ. Establishing human leukemia xenograft mouse models by implanting human bone marrow-like scaffold-based niches. Blood (2016) 128(25):2949–59. doi: 10.1182/blood-2016-05-719021 27733356

[90] Friedrich C Kosmider O. The mesenchymal niche in myelodysplastic syndromes. Diagn (Basel Switzerland) (2022) 12(7):1639. doi: 10.3390/diagnostics12071639 PMC9320414 35885544

